# Incipient Stages of Scabies from Sarcoptes Canis

**Published:** 1883-07

**Authors:** 


### VI.—INCIPIENT STAGES OF SCABIES FROM SARCOPTES CANIS.

REPORTED FROM DR. STICKLER'S CLINIC AT THE COLLEGE.

The patient is a black bitch (pointer) the property of Prof. Laudy. She was sent to the hospital December 18th. About three weeks ago she began to lose hair, in spots about the size of a ten-cent piece, on face and body, which was accompanied with a slight amount of itching; these spots gradually increasing in size.

The above diagnosis was given, and a favorable prognosis rendered.

*Treatment.*

The following ointment to be used every half hour, at first; later on, less frequently;

℞

| | |
|---|---|
| Adeps, . . . | Oi. |
| Sulph. Sublimat, . | ℥iiss. |
| Sodæ Bicarb., . . | ʒvi. |

♏ Sig. ex. use.

An Anthelmintic was given in the shape Oleum Santonis ♏ ii— that the worms might be destroyed.

